# Extracellular Vesicle-Based Characterization of Stem Cell Phenotype in Glioblastomas

**DOI:** 10.7759/cureus.70403

**Published:** 2024-09-28

**Authors:** Georgiana M Serban, Manu Doina, Rodica Balasa, Adrian F Balasa

**Affiliations:** 1 Doctoral School, “George Emil Palade” University of Medicine, Pharmacy, Science, and Technology of Targu Mures, Targu Mures, ROU; 2 Anesthesiology and Critical Care, Emergency Clinical County Hospital of Targu Mures, Targu Mures, ROU; 3 Biological Sciences, “George Emil Palade” University of Medicine, Pharmacy, Science, and Technology of Targu Mures, Targu Mures, ROU; 4 Neurology, “George Emil Palade” University of Medicine, Pharmacy, Science, and Technology of Targu Mures, Targu Mures, ROU; 5 Neurosurgery, Emergency Clinical County Hospital Of Targu Mures, Targu Mures, ROU

**Keywords:** cd133, cd326/epcam, cd44, extracellular vesicles, glioblastoma, nanogp8, ssea4

## Abstract

Glioblastoma (GB) is the most common brain malignancy occurring in adult patients having an extremely low overall survival. Therefore, it is paramount to establish reliable and accurate diagnostic and prognostic markers to guide a personalized and more effective treatment. Molecular characterization of the tumor is the ultimate goal in GB management and comprises, among others, the study of the extracellular vesicles (EVs). Not only do they carry within their cargo molecules involved in shaping a favorable microenvironment for GB development, but EVs also present surface markers mirroring the phenotype of the donor cells. Our study aims to assess the dynamic evolution of EV-positive surface biomarkers and EV-derived proteins involved in maintaining and transferring a stem cell phenotype to the cells from GB surroundings. We performed a prospective observational study on GB patients operated on in the Neurosurgery Clinic of the Emergency Clinical County Hospital of Târgu Mureș, Romania. GB-derived EVs were isolated from the patients’ plasma using a density gradient ultracentrifugation protocol. The expression of EVs positive to four epitopes specific to stem cells (CD44, CD133, CD326/EpCAM, and SSEA4) was followed in three moments in time, preoperatively, seven days, and three months postoperatively, respectively, and quantified by a bead-based multiple analysis using flow cytometry. Moreover, NANOGP8, a protein within GB cargo capable of promoting a stem cell phenotype, was dynamically evaluated using the Western blot technique. Our study showed a statistically significant decrease of all surface markers and NANOGP8 immediately after tumor ablation. Nonetheless, the long-term follow-up of the patients revealed an extremely variable evolutionary pattern reflecting the high heterogeneity of GB. Further studies are necessary to either confirm or infirm the accuracy of these markers in early diagnosing GB, in predicting the outcome of this disease, and in guiding an individualized therapy.

## Introduction

Glioblastoma (GB) is the most frequent and the most aggressive primary malignancy of the central nervous system occurring in adult patients [1]. In spite of a rapid diagnostic and therapeutic approach, it still has a horrendously low overall survival and an unfavorable prognosis [2]. As a result, the late research focus has been on establishing accurate and efficient diagnostic and prognostic markers to guide a personalized and, hopefully, much more effective treatment. Various factors related to the patient (e.g., age at the moment of diagnosis, preoperative clinical status), to the tumor itself (e.g., localization, volume, histopathological and molecular subtype), to the treatment (e.g., the extent of surgical ablation, response, and tolerance to adjuvant therapy), and even to the body immune-inflammatory response to the malignancy have been taken into consideration [3,4]. Unfortunately, none of these has proven to be reliable enough to predict the evolution of the disease, most probably due to the intra- and intertumoral heterogeneity leading to a huge variability in the patients’ response to therapy [5].

Extensive research into GB pathogenesis emphasized the role of biomarkers derived from circulating tumor cells, nucleic acids coding material, metabolites, and extracellular vesicles (EV) [6]. EVs are nanostructures derived from both normal and tumoral cells. They carry a vast variety of molecules within their cargo that are specific to the donor cells and, consequently, mirror the GB behavior [7]. EVs are involved in shaping an optimal microenvironment for tumoral thriving by stimulating GB progression, invasion, and migration, inducing radiotherapy and pharmacological resistance, and enhancing angiogenesis [2]. Due to specific EV structure permitting the blood-brain barrier passage, it is currently possible not only to isolate and characterize EVs but also to quantify the molecules within their cargo using minimally invasive techniques [8].

One of the processes underlying the distinct GB aggressivity is the maintenance of a stem cell phenotype associated with indefinite cellular division and self-renewal [9]. EVs facilitate the transfer of this feature among tumor cells and from tumor cells to previously healthy cells. Among other molecules transported within EV cargo, NANOGP8 has been suggested as an important promoter of cancer stemness in different malignancies, GB included [9]. Moreover, the EVs carry surface markers specific to cancer stem cells, namely, CD44, CD133, SSEA4 (stage-specific embryonic antigen-4), and CD326/EpCAM (epithelial cell adhesion molecule) that allows their identification and characterization [10,11].

In this pilot study, we aimed to assess the dynamic trend of NANOGP8 released by GB-derived EVs in three key moments, namely, preoperatively, seven days, and three months after surgery, and to establish its potential diagnostic and prognostic role in GB patients. Furthermore, we sought to establish an evolutionary pattern for CD44, CD133, SSEA4, and CD326/EpCAM positive EVs to predict GB outcome.

## Materials and methods

Patient selection

We conducted a prospective, observational study during a 42-month period from January 2021 until end of June 2024 in the Neurosurgery Clinic of the Emergency Clinical County Hospital of Târgu Mureș, Romania. The study was previously approved by the Local Ethics Committee of the Emergency Clinical County Hospital of Târgu Mureș (decision no. 29573/08.12.2020). The laboratory experiments were performed in the Immunology Laboratory of the Advanced Medical and Pharmaceutical Research Center within the "George Emil Palade" University of Medicine, Pharmacy, Science, and Technology, Târgu Mureș Romania, in accordance with the Declaration of Helsinki principles regarding medical research on human. Prior to clinical and imagistic data and blood sample collection and analysis, each patient filled in and signed a written informed consent. We included in our study the adult patients who were diagnosed with primary GB and underwent surgical removal of the tumor in our clinic between January 2021 and December 2022, as well as socially and demographically matched non-cancerous controls. All our patients received postoperative radiotherapy and pharmacological treatment with temozolomide according to the Stupp protocol. The exclusion criteria were histopathologically confirmed secondary GB, refusal of surgery, prior history of neoplasia, and/or chemo-/radiotherapy. We clinically evaluated our subjects in three key moments, which are preoperative (T1), seven days (T2), and three months (T3) after surgery, when we also collected peripheral blood samples.

We summarized the patients’ data stored within the Hospital Information System and those obtained from the patients and their relatives or legal representatives, such as gender, age at the moment of diagnosis, tumor properties (e.g., localization, dimension - the largest measurement in the axial plane on the MRI examinations expressed in millimeters, histopathological features), and clinical status quantified by Karnofsky Performance Scale (KPS) in the three key moments.

EV isolation from peripheral blood

Peripheral blood was collected from the non-cancerous controls and from GB patients in the three aforementioned moments in 9-mL K2-EDTA vacutainers (Becton Dickinson, Franklin Lakes, NJ, USA) and further processed within two hours by two-step centrifugations: at 300xg for 10 minutes at 4°C to obtain plasma and at 2000xg for 20 minutes at 4°C to remove the platelets and cellular debris. The plasma was frozen at -80°C.

**Figure 1:**
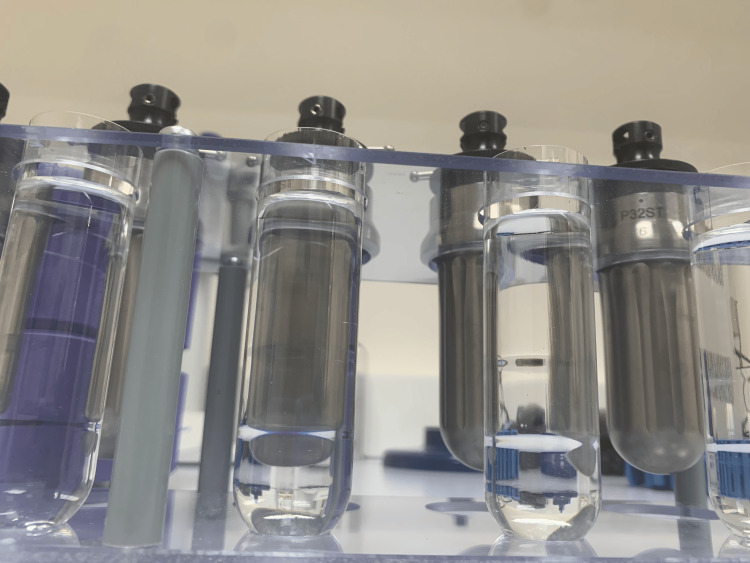
Serial dilutions of iodixanol – OptiPrepTM in Tris-HCl buffer enriched in sucrose 0.25 M in order to obtain four layers of different concentrations and densities for extracellular vesicle (EV) isolation based on the density gradient ultracentrifugation method.

The density gradient ultracentrifugation method (DGU) was used to isolate the EVs from the GB patients’ blood with CP100NX ultracentrifuge equipped with Hitachi P23ST rotor. The density gradient was obtained by serial dilutions of iodixanol - OptiPrepTM in Tris-HCl buffer enriched in sucrose 0.25M. Thus, four layers of different concentrations and densities were achieved: 40% - 1.255 g/cm^3^, 20% - 1.150 g/cm^3^, 10% - 1.097 g/cm^3^, and 5% - 1.065 g/cm^3^ (Figure 1).

**Figure 2:**
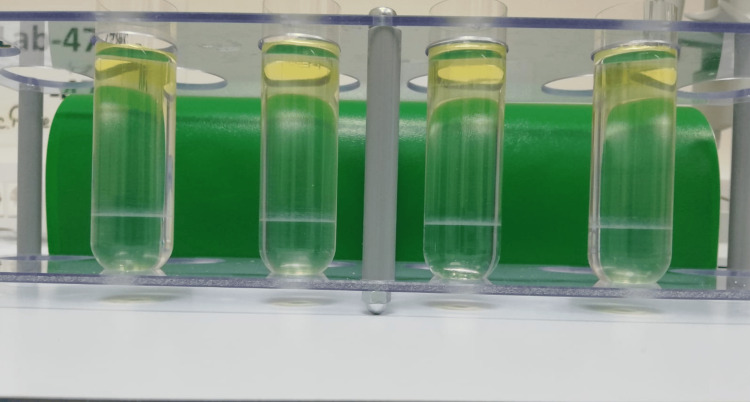
Ultracentrifugation-induced extracellular vesicle (EV) migration between the 40% and 20% dilution visualized as an opaque ring at that level.

The thawed plasma was added on the top of the four layers and ultracentrifuged at 24000 rpm for 18 hours at 4°C. The EVs, containing the exosomes, the apoptotic bodies, and the microvesicles, were fractioned in the density gradient according to their specific density. The exosomal density is recognized to be between 1.15 and 1.19 g/cm^3^, and, therefore, the exosomes gathered as an opaque ring at the border between the 40% and 20% dilution (Figure 2).

A second ultracentrifugation at 24000 rpm for one hour at 4°C was performed in order to increase the suspension's purity.

Bead-based multiplex EV analysis by the flow cytometry method

**Figure 3:**
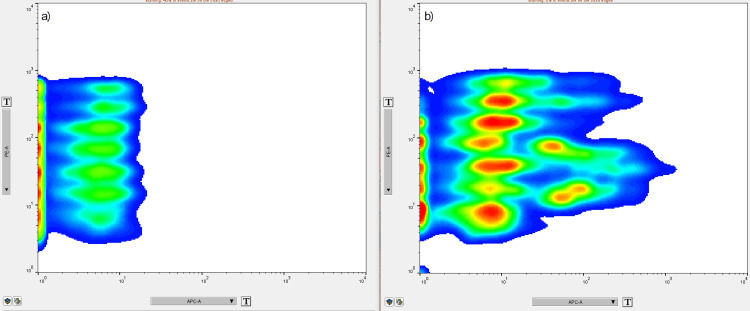
a) APC-negative buffer control; b) APC-positive, tetraspanin (CD9, CD63, and CD81) - positive capture bead populations.

EVs were captured on antibody-coated beads (MACSPlex Exosome Capture Beads) from MACSPlex Exosome Kit (Miltenyi Biotec, catalog no. 130-122-209) that could be distinguished by different fluorescence intensities in the FITC and PE fluorescence channels. EVs bound by the capture beads were further stained with APC-conjugated anti-CD9, anti-CD63, and anti-CD81 antibodies (MACSPlex Exosome Detection Reagents). CD9, CD3, and CD81, also called tetraspanins, are surface markers specific to exosomes. Tetraspanin-positive EVs and CD44, CD133, CD326/EpCAM, and SSEA4-positive EVs were detected with BD FACSAria III flow cytometer using BD FACSDivaTM v 8.01 software (Figure 3).

**Figure 4:**
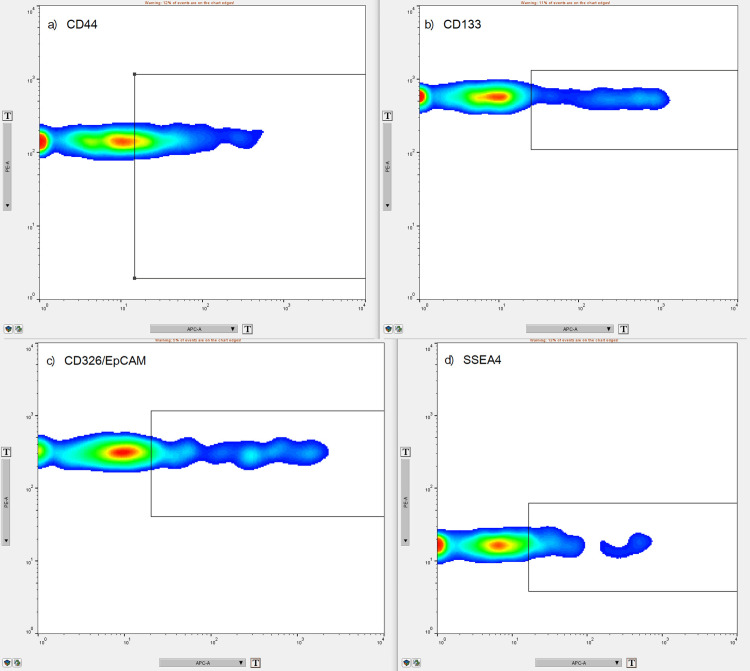
FlowJo representation of gating strategy for APC-positive markers based on normalization with corresponding negative control: a) APC-positive CD44, b) APC-positive CD133, c) APC-positive CD326/EpCAM, d) APC-positive SSEA4.

A volume of 120 μL from each EV suspension sample and from the buffer (as negative control) was used to detect EVs. The acquired data were exported in FlowJo v10 software as .fcs files. The mean fluorescence intensity of each sample normalized with the negative control was used for further statistical analysis (Figure 4).

Western blot quantitative analysis of EV proteins

Western blot analysis on the EV suspensions obtained by DGU was used to detect and quantify NANOGP8 within the EV cargo. EV suspensions were lysed with an equal volume of ice-cold phenylmethylsulfonyl fluoride (PMSF, Abcam, catalog no. ab141032) enriched RIPA buffer (Abcam, Cambridge, UK, catalog no. ab156034). After one-step centrifugation at 14000xg for 15 minutes at room temperature, the lysate was mixed with an equal volume of 2× Laemmli Sample Buffer (Bio-Rad Laboratories, Hercules, CA, USA, catalog no. 1610737) and with 5% β-mercaptoethanol (Bio-Rad Laboratories, catalog no. 1610710) and subjected to denaturation for five minutes at 95°C.

The EV-derived proteins were electrophoretically separated using SDS-PAGE (sodium dodecyl-sulfate polyacrylamide gel electrophoresis) with tris/glycine/SDS (TBS) running buffer (Bio-Rad Laboratories, catalog no. 1610732) in the Mini-PROTEAN® Tetra Vertical Electrophoresis Cell System (Bio-Rad Laboratories, Hercules, CA, USA).

The separated proteins were then transferred to polyvinylidene difluoride (PVDF) membranes using the Trans-Blot® Turbo™ Transfer Pack (Bio-Rad Laboratories, catalog no. 1704156) with the Trans-Blot® Turbo™ Transfer System (Bio-Rad Laboratories, Hercules, CA, USA). The membranes were next treated with TBST (TBS+0.1% Tween® 20) + BSA (bovine serum albumin) 3% blocking buffer in order to diminish non-specific binding. EV-derived NANOGP8 was detected through overnight incubation with specific primary rabbit anti-human NANOGP8 antibody (Invitrogen NANOGP8 Polyclonal Antibody, catalog no. PA5-99659), followed by one-hour incubation with peroxidase-conjugated secondary goat anti-rabbit antibody (Invitrogen Goat anti-Rabbit IgG (H+L) Cross-Adsorbed Secondary Antibody, HRP, catalog no. G-21234). The chemiluminescent detection in the presence of luminol (Clarity™ Western ECL Substrate kit, Bio-Rad Laboratories, catalog no. 1705061), image evaluation, and band intensity quantitative analysis were performed using ChemiDoc XRS+ System (Bio-Rad Laboratories, Hercules, CA, USA) and ImageLab™ software version 6.1.0 (Bio-Rad Laboratories, Hercules, CA, USA).

Statistical analysis

The data were statistically analyzed using GraphPad software. According to the Kolmogorov-Smirnov normality test, we evaluated the distribution of our numerical data and further presented them either as mean ± standard deviation (in the case of Gaussian distribution) or as median ± interquartile range (in the case of a non-Gaussian distribution). Related to parametrically distributed paired data, we used variants of Student's t-test to compare two sets of data and variants of the ANOVA test to compare three or more samples, respectively. For paired data with non-Gaussian distribution, we applied the Wilcoxon test for two samples and the Friedman test for three or more sets of data, respectively. In the case of statistically significant results when comparing three or more samples, we further applied a post-hoc test to reliably control the type 1 error rate: either the Tukey test for parametrically distributed data or the Dunn test for non-parametrically distributed data. We also analyzed potential correlations between two sets of data using either Pearson’s or Spearman’s correlation coefficient in relation to data distribution. A p-value lower than 0.05, with a 95% confidence interval, was considered statistically significant. 

## Results

According to the inclusion and exclusion criteria, our study initially comprised 24 patients at T1, 15 men (62.5%) and nine women (37.5%), with a mean age of 60.5 years old. One female patient died five days postoperatively, so 23 blood samples were collected at T2. Only nine blood samples were obtained at T3. The preoperative clinical status of the patients was quantified by the Karnofsky Performance Scale (KPS) and the recorded T1 median was 80. The patient reported dead was the most severely affected preoperatively, with a KPS of 20. Only two patients are still alive. All our patients underwent surgical intervention with total tumor ablation (macroscopically), and 23 out of 24 further followed adjuvant therapy with temozolomide and radiotherapy, according to Stupp protocol.

At the moment of diagnosis, the mean size of the tumor was 47.35 ± 14.79 mm. There was a slight left predominance of the tumor localization (54.16% vs. 45.83%). The parietal (n = 9) and temporal (n = 8) regions were the most frequently interested, while the frontal (n = 4) and occipital (n = 3) ones were the least affected in our population (Table 1).

**Table 1:** Demographical, clinical, and tumor-related characteristics of the glioblastoma (GB) patients. M - male, F - female, KPS - Karnofsky Performance Status Scale, T1 - preoperative moment

Patient	Age	Gender	Localization	Tumor side	Dimension (mm)	KPS (T1)
G1.1	59	M	Parietal	Right	48	90
G2.1	65	M	Parietal	Right	20	90
G6.1	68	M	Frontal	Right	43	80
G9.1	57	M	Parietal	Right	42	90
G10.1	74	M	Parietal	Right	71	80
G13.1	55	F	Temporal	Left	29	90
G14.1	49	M	Temporal	Left	59	80
G15.1	67	F	Occipital	Right	37	90
G16.1	76	F	Parietal	Left	37	80
G20.1	51	F	Temporal	Right	40	60
G21.1	44	F	Frontal	Left	59	80
G23.1	67	F	Frontal	Right	34	80
G25.1	79	F	Parietal	Left	40	80
G26.1	71	M	Temporal	Right	66	80
G27.1	46	F	Occipital	Left	80	20
G28.1	57	M	Temporal	Left	53	80
G32.1	60	F	Temporal	Left	36	90
G36.1	48	M	Occipital	Right	50	90
G37.1	47	M	Parietal	Right	47	90
G38.1	62	M	Frontal	Left	54	80
G39.1	71	M	Temporal	Left	38	90
G41.1	59	M	Temporal	Left	54	80
G42.1	68	M	Parietal	Left	70	80
G43.1	54	M	Parietal	Left	30	90

**Figure 5:**
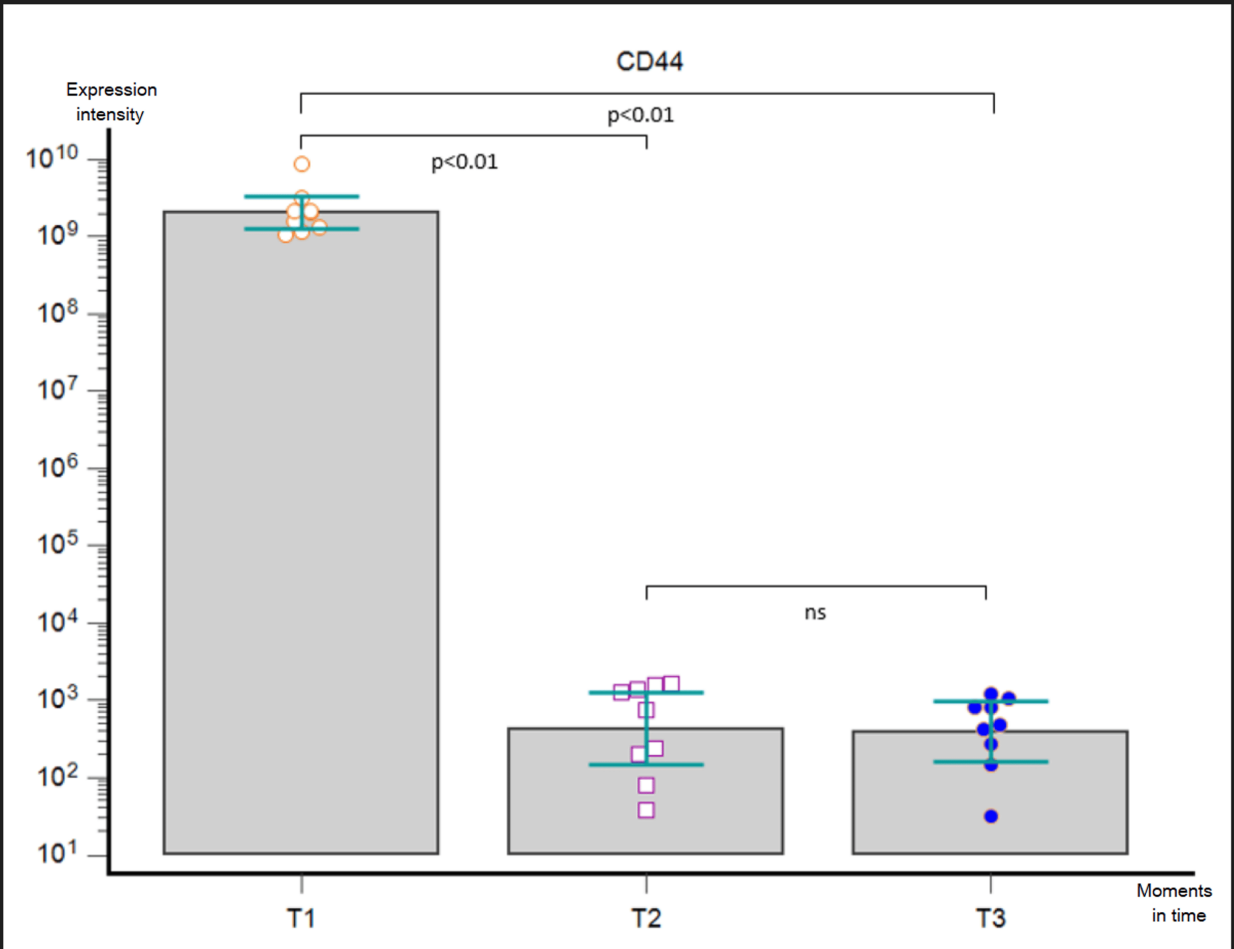
Friedman’s ANOVA analysis corrected by Dunn’s post-hoc test performed on GB patients in the three moments (T1, T2, and T3) for EV-derived CD44 expression. ns - statistically non-significant, ANOVA - analysis of variance, GB - glioblastoma, EV - extracellular vesicle

**Figure 6:**
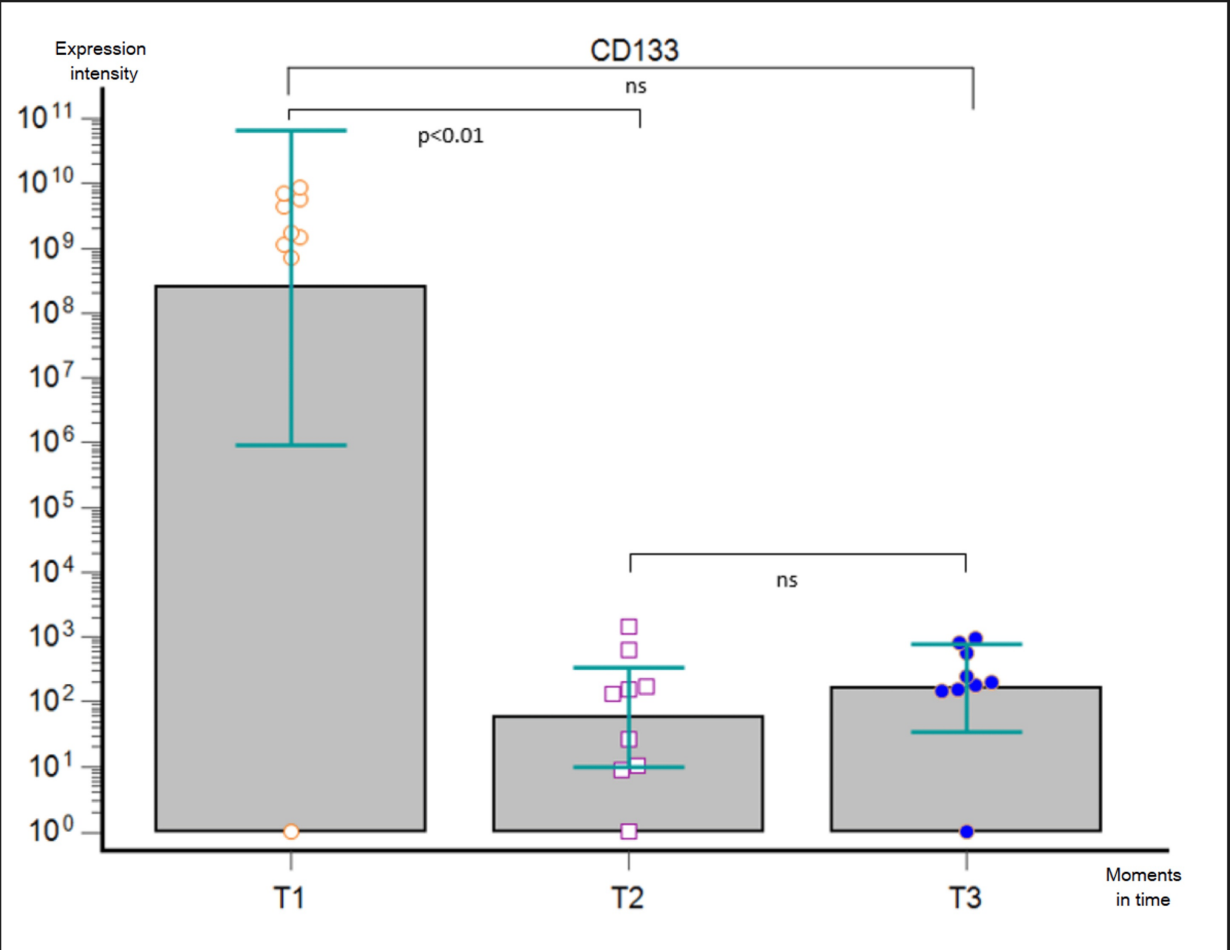
Friedman’s ANOVA analysis corrected by Dunn’s post-hoc test performed on GB patients in the three moments (T1, T2, and T3) for EV-derived CD133 expression. ns - statistically non-significant, ANOVA - analysis of variance, GB - glioblastoma, EV - extracellular vesicle

**Figure 7:**
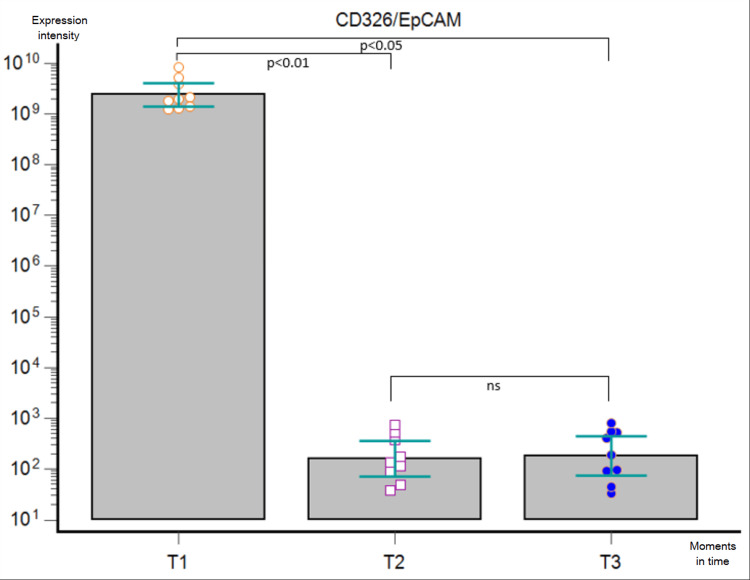
Friedman’s ANOVA analysis corrected by Dunn’s post-hoc test performed on GB patients in the three moments (T1, T2, and T3) for EV-derived CD326/EpCAM expression. ns - statistically non-significant, ANOVA - analysis of variance, GB - glioblastoma, EV - extracellular vesicle

**Figure 8:**
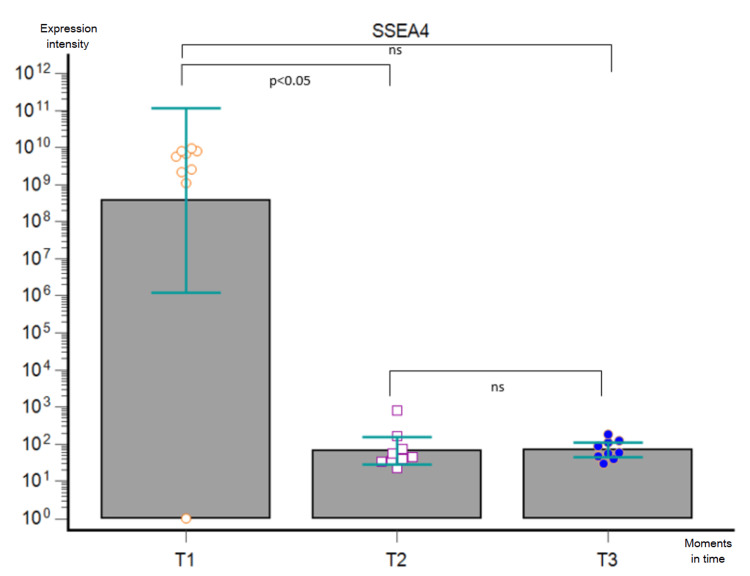
Friedman’s ANOVA analysis corrected by Dunn’s post-hoc test performed on GB patients in the three moments (T1, T2, and T3) for EV-derived SSEA4 expression. ns - statistically non-significant, ANOVA - analysis of variance, GB - glioblastoma, EV - extracellular vesicle

We performed a bead-based multiplex analysis using the flow cytometry technique and MACSPlex Exosome Kit and quantitively assessed the dynamic expression of CD44, CD133, CD326/EpCAM, and SSEA4 for each patient at T1, T2, and T3, compared to non-cancerous controls. The acquired data were extremely heterogeneous, with great variability not only among the patients at one particular moment but also among the dynamic trends recorded in the aforementioned three key moments. When comparing the marker levels in T1 vs. T2, we obtained a statistically significant postoperative decrease of every marker (p < 0.0001). Furthermore, Friedman’s ANOVA analysis applied for each surface marker individually showed a statistically significant difference between the three moments in time (CD44 - p = 0.0003, CD 133 - p = 0.0067, CD326/EpCAM - p = 0.0002, and SSEA4 - p = 0.0103, respectively): their expression lowered after surgical removal of the tumor, but it was slowly increasing three months later. Nonetheless, post-hoc Dunn test confirmed the statistically significant differences between T1 and T2 for all markers and between T1 and T3 for CD44 and CD326/EpCAM, whereas it showed no statistically significant differences between T2 and T3 for either marker and between T1 and T3 for CD133 and SSEA4 (Figures 5-8).

Furthermore, NANOGP8 data are summarized in Table 2

**Table 2:** Quantitative expression of NANOGP8 protein in GB patients in T1, T2, and T3. T1 - preoperative moment, T2 - seven days postoperative, T3 - three months postoperative

NANOGP8	T1	T2	T3
G1	10255775	5888646	-
G2	8109036	4864320	10491470
G6	10960326	7322292	-
G9	8573214	3029325	1759698
G10	6211504	6440025	-
G13	16972620	1652025	-
G14	16827772	1698150	0
G15	19635668	2793325	5415660
G16	12966070	1429400	0
G20	5558640	3821850	-
G21	2392992	5328199	12449754
G23	4106500	5522322	-
G25	3063925	4136640	-
G26	1516242	2361488	-
G27	3226550	-	-
G28	2951496	15921757	-
G32	1047982	1676280	5617512
G36	4380376	3762065	0
G37	2151792	1684539	0
G38	3033936	950317	-
G39	3574530	765181	-
G41	3210786	3322836	-
G42	3930282	353092	-
G43	12133800	553257	-

**Figure 9:**
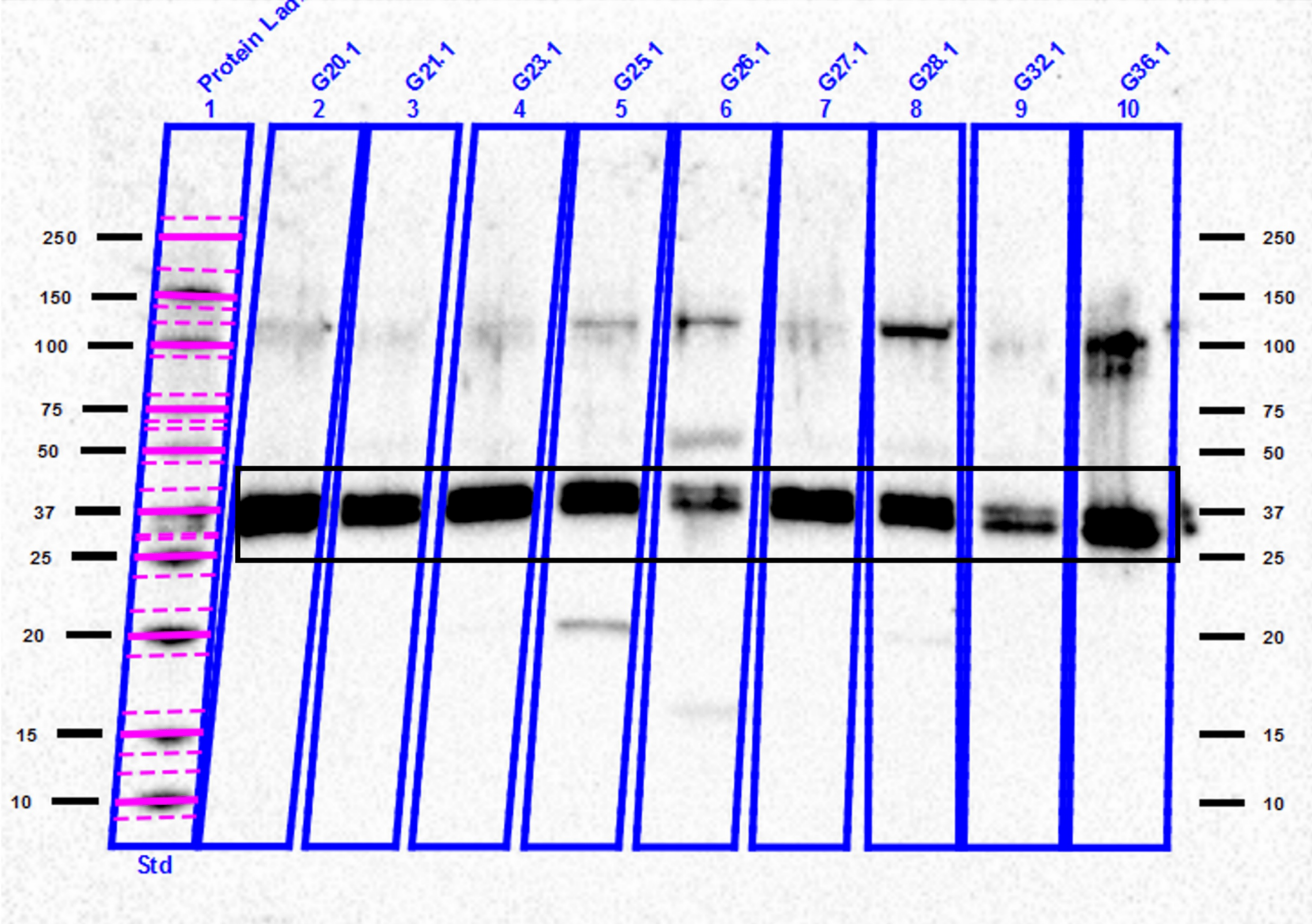
Representative blots of GB-derived EVs aliquots with anti-NANOGP8 antibody at T1. NANOGP8 bands were observed at approximately 35 kDa. Additional bands at 100-180 kDa were noticed in almost all patients. GB - glioblastoma, EV - extracellular vesicle

**Figure 10:**
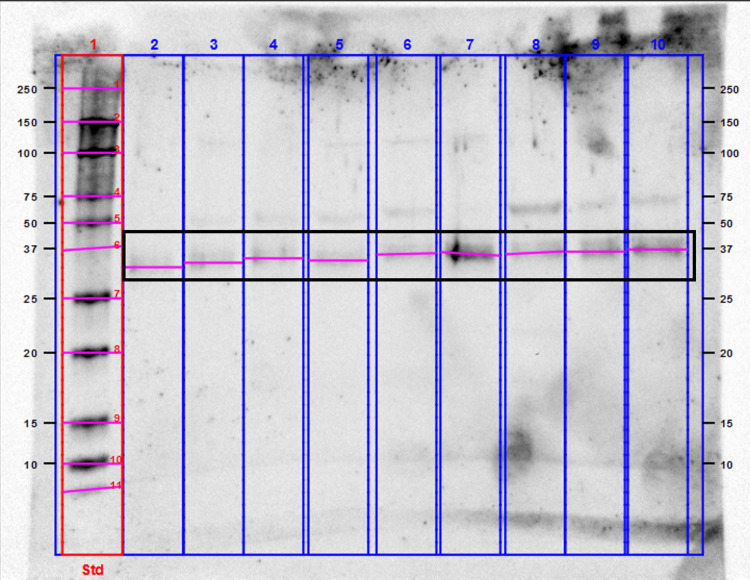
Representative blots of GB-derived EV aliquots with anti-NANOGP8 antibody at T2. NANOGP8 bands were observed at approximately 35 kDa. Additional bands at 80-180 kDa were noticed in almost all patients. GB - glioblastoma, EV - extracellular vesicle

We detected bands of NANOGP8 protein at approximately 35 kDa. We also observed additional bands between 100 and 150 kDa in most of the GB patients, which may be interpreted as the presence of different conformations of NANOGP8 with distinct electrophoretic mobility related to cryopreservation at -80°C [12] (Figures 9, 10).

**Figure 11:**
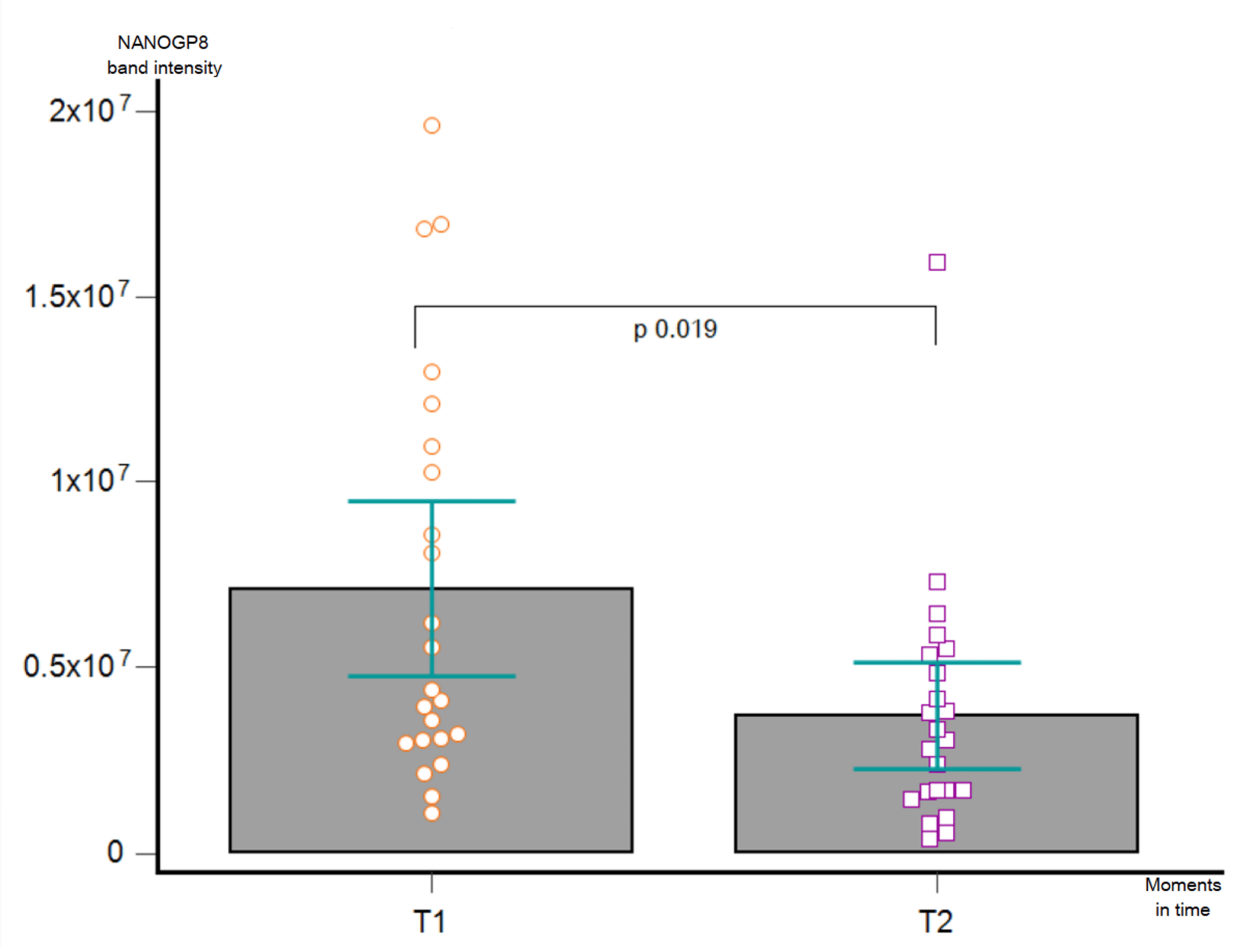
Wilcoxon match-paired test applied to compare NANOGP8 protein expression in T1 vs. T2.

We assessed the protein amount in both controls and GB patients. NANOGP8 showed no detectable bands in control samples. The amount of NANOGP8 is significantly more reduced at T2 in comparison with T1 (p = 0.019) (Figure 11).

**Figure 12:**
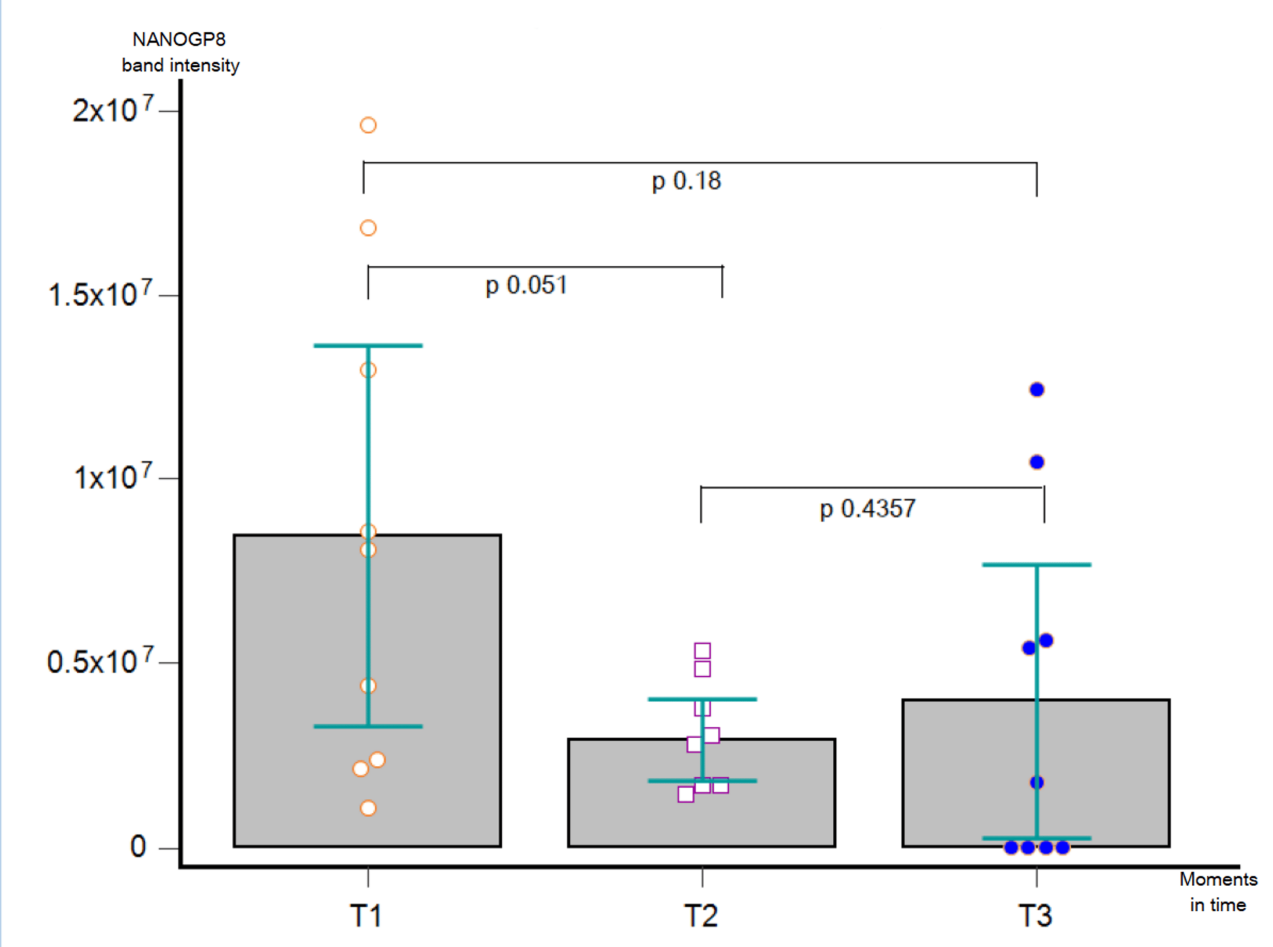
ANOVA repeated-measures analysis applied to compare the NANOGP8 expression in T1 vs. T2 vs. T3, p = 0.0735. No post-hoc test has been further applied as a statistically non-significant result had been obtained.

When assessing the dynamic trend of NANOGP in the nine patients from T3, the ANOVA analysis showed no statistically significant results (p = 0.0735). Nonetheless, the same diminution in the NANOGP8 quantity was obtained in T2 vs. T1 (p = 0.051). The heterogeneous data at T3 were compared to both T1 (p = 0.18) and T2 (p = 0.4357). Some patients had very high protein amounts at T3, even higher than at T1, while other patients showed no detectable bands of NANOGP8 at T3 (Figure 12).

Moreover, we assessed the potential correlations between preoperative NANOGP8 and tumor dimensions at the moment of diagnosis (p = 0.07, Spearman r = -0.3699) and between NANOGP8 and KPS at T1, T2, and T3, yet no statistically significant results have been obtained.

## Discussion

The latest focus in GB research is on finding the most appropriate diagnostic and prognostic tools to guide a more personalized therapy in order to increase overall survival. The EVs have a recognized role in tumorigenesis by modulating tumoral proliferation, invasion, migration, immunosuppression, and supportive angiogenesis through their involvement in intercellular communication [2]. Moreover, they carry various surface markers and numerous molecules within their cargo that are specific to the donor cells exhibiting their functional status [13]. Nonetheless, there is still no generally accepted EV marker to guide GB treatment, and considerable efforts are being made to establish one [13].

Our study has focused on the EV markers with known roles in maintaining and transferring a stem cell phenotype from tumor cells to previously healthy cells. Cellular stemness confers huge aggressiveness to GB not only by facilitating unlimited proliferation but also by increasing the resistance to chemo- and radiation treatment, eluding the immune response and favoring recurrences [14,15]. NANOG is an important transcriptional factor that regulates embryonic stemness in mammalian cells: the more differentiated the cells are, the lower amounts of NANOG they display [15]. NANOGP8 derives from NANOG by retrotransposition and encodes a full-length nuclear protein capable of promoting stemness [16]. Vaidya et al. [17] have demonstrated that NANOGP8 is more expressed In GB cancer cells and cancer stem cells (CSCs) than in normal neural stem cells. Our study showed a significant reduction of NANOGP8 amount at T2 compared to T1, which could be explained by the surgical ablation of the tumor with consequent diminished tumor mass and cancer cells and CSC number. Surprisingly, we found a negative, yet not statistically significant, correlation between the preoperative GB size and NANOGP8 level at T1. This contradictory result might be justified by using the largest tumor dimension in the axial plane and not the entire tumor volume, which in reality could be higher in spite of a smaller one-plane measurement.

On the other hand, the long-term follow-up of NANOGP8 levels (at three months postoperatively) showed an extremely variable trend. There were patients with MRI-proven recurrence with no detectable bands of NANOGP8 protein at T3 (e.g., G14), whereas there were subjects with no macroscopically apparent tumor having a high level of NANOGP8 (e.g., G15). Only one patient (G36) with blood samples at T3 is still alive, who had no NANOGP8 recorded three months postoperatively. On the one hand, the presence of NANOGP8 in cases with no apparent recurrences could be explained by the limited ability of diagnostic tools to detect early recurrences [18]. Despite the insignificant proportion of CSCs within the whole GB environment, they are extremely protean and capable of modifying their behavior in order to survive to changing environment [18]. As a result, even a macroscopically undetectable tumor could release a significant amount of NANOGP8-enriched EVs. On the other hand, according to Vaidya et al. [15], NANOGP8 expression is deeply dependent on tumor microenvironment and on oncogenic transcriptional factors that may or may not enhance the activation of specific promoters in NANOGP8 DNA sequences. Furthermore, postoperative necrosis could be easily confounded with a GB recurrence [19]. Only the histopathological examination could clarify this dilemma, but our patient did not undergo another surgical intervention.

The recognized molecular weight of NANOGP8 is approximately 35 kDa when migrating on SDS-PAGE gels. Nevertheless, our results showed additional bands at molecular weights varying between 25 kDa and 180 kDa. Liu et al. [12] have debated this issue and concluded that either alternative splicing or intrinsic biochemical properties of NANOGP8 could lead to migration at a broader range of molecular weights from 30 to 180 kDa. In addition, long-term storage at -80°C could result in heavier molecules and a consequent slower migration [12].

We also performed a bead-based multiplex analysis, which quantified the surface markers on the EVs. We aimed to establish a general evolutionary pattern allowing to minimally invasively diagnose even microscopic tumors and to predict the outcome of GB patients. We obtained instead patient-specific signatures, which is not surprising considering the huge intra- and intertumoral heterogeneity of GBs [18]. We concentrated our analysis on CD44, CD133, SSEA-4, and CD362/EpCAM in addition to NANOGP8, as all are deeply involved in inducing a stem cell phenotype [20,21]. Although the ANOVA analysis has shown a statistically significant difference between the three moments in every surface marker, the post-hoc Dunn test infirmed the statistical significance particularly for the expression of surface markers at T3 in comparison with T2. Moreover, we could observe the huge differences among the dynamic trends for each individual patient.

The glial stem cells (GSCs) represent a subpopulation in the tumor microenvironment whose essential role in GB aggressiveness is due to their stemness abilities. Although they usually have CD133 as their surface marker, studies have shown that CD133 expression depends on tumor microenvironment response to stressful conditions, and thus, there are CD133-negative GSCs as well [22]. In addition, CD133 is not specific to GB stem cells, as they are expressed in other stem cells and in tumor cells of different origins [22]. Nonetheless, Bien-Möller et al. [22] demonstrated a significant association between CD133 level and GB patients’ survival: high CD133 expression relates to an unfavorable prognosis, particularly due to increased refractoriness to both chemo- and radiotherapy. According to Zhang et al. [23], CD133+ GB-derived CSC development is dependent on NANOGP expression. Moreover, Meng et al. [24] showed that NANOGP8 expression is stronger in CD133+ colorectal tumor cells. Our study demonstrated a significant decrease in CD133-positive EVs at T2 when compared to T1, most probably explained by the reduced tumor mass consequent to surgical removal. Our results revealed an apparent, yet not statistically significant, increase at T3, probably related to GB recurrences.

CD44 is a transmembrane glycoprotein that modulates cancer stemness and metastases and promotes proliferation and invasion [25]. It is expressed on the membrane of GB CSCs and it is related to poor survival and prognosis [25]. The alternative splicing of the CD44 gene leads to multiple CD44 molecular variants with individual functions [10]. Three isoforms have been identified in GB so far, but only one (CD44v6) plays a demonstrated role in GSC growth modulation [10]. CD44 consists of three domains, among whom the intracellular cytoplasmic domain (ICD) is the one that intermediates the interplay between CD44 and NANOGP8 [10]. CD44-ICD stimulates hypoxia-induced HIF-2α pathway that further activates HIF-2α-target genes, NANOG included. This molecular route is more expressed in CSCs than in differentiated tumor cells and is deeply involved in maintaining a stem cell phenotype [26]. Our GB patients had much more marked expressions of CD44-positive EVs at T1 in contrast with both T2 and T3. However, a significant difference between seven days and three months postoperatively was not demonstrated.

EpCAM/CD326 is a multifunctional transmembrane glycoprotein that participates in tumor proliferation, metabolism, immunosuppression, treatment resistance, angiogenesis, and, last but not least, cancer stemness [21]. Zhang et al. [26] have proposed a molecular pathway involving both EpCAM and NANOG to increase the aggressiveness of breast malignancies. It seems that under hypoxic circumstances, EpCAM overexpression stimulates the occurrence of stemness representatives, such as NANOG, in a way dependent on hypoxia-induced factor 1α and nuclear factor κB [26]. Furthermore, the co-expression of NANOG and EpCAM is mandatory to succeed in converting somatic cells into pluripotent stem cells [27]. Similarly, with the previous surface marker, CD326/EpCAM-positive EVs generated noticeable signals at T1 in comparison with both T2 and T3.

SSEA-4 is a glycosphingolipid recognized as a pluripotency marker and utilized for characterization of the cells with stemness ability [28]. Moreover, Yasmin et al. [29] have demonstrated the presence of both NANOG and SSEA-4 in GB cells originating from induced pluripotent stem cells. Our study demonstrated an increased expression of SSEA4-positive EVs at T1 when compared to T2. No other statistically significant results were obtained.

Study limitations

The main limitation of our pilot study is the reduced number of patients, especially at T3. This is explained not only by the single-center implementation but also by the lack of standardization of a reproducible protocol for EV isolation and characterization. We opted for the DGU isolation and Western blot characterization techniques; however, they are time-consuming and difficult to implement in a larger cohort. Moreover, the aggressive nature of the disease and the side effects of the adjuvant therapies made the follow-up of the patients and, consequently, the transportation to the hospital for collecting the blood samples too difficult for most of them, especially for those living at considerable distances from our hospital.

Furthermore, as far as we are concerned, this is the first study to quantify the NANOGP8 protein within the cargo of the GB-derived EVs from peripheral blood. We cannot exclude the presence of NANOGP8 protein from other sources. Nonetheless, as our controls showed no trace of NANOGP8 protein, we are inclined to believe that the results we obtained are primarily of GB origin. 

Moreover, most of the research involving surface markers of stemness is directly performed on tumor-derived cells. Our study focused on EVs released by GB-related cells, which mirror the behavior of the donor cells. Nonetheless, whether quantitative analysis of EV surface markers and cellular epitope expression shows overlapping results is still difficult to conclude. 

## Conclusions

The milestone in GB management is finding accurate and effective diagnostic and prognostic markers to guide the treatment. It is clear that the molecular characterization of the tumor, including GB-derived EV analysis, should be the current focus. The EV cargo contains numerous molecules involved in different processes that favor tumor development, among which the stem cell phenotype is recognized. NANOGP8 is one of the main representatives of tumoral stemness. Our study proved that a total tumor ablation is associated with reduced NANOGP8 values in the short term (one week postoperatively), yet it failed to demonstrate a concrete trend in the long term (at three months postoperatively). Furthermore, EVs exhibit surface markers that reflect the stemness ability of the secreting cells, such as CD44, CD133, CD326/EpCAM, and SSEA4. Their expression dramatically decreases after tumor ablation; however, the long-term evolution is extremely variable among patients. Further studies are, thus, necessary to confirm or infirm NANOGP8, CD44, CD133, CD326/EpCAM, and SSEA4 as diagnostic and/or prognostic biomarkers in GB patients.
